# Representation of Erie County Medical Society, and University of Buffalo, in New York State Medical Society

**Published:** 1864-12

**Authors:** 


### EDITORIAL DEPARTMENT.

### REPRESENTATION OF ERIE COUNTY MEDICAL SOCIETY, AND UNIVERSITY OF BUFFALO, IN NEW YORK STATE MEDICAL SOCIETY.

We received the volume of the Transactions of the New York State Medical Society with the following note from the Secretary:

"MEDICAL SOCIETY OF THE STATE OF NEW YORK,
Albany, N, Y., October 22, 1864.

DR. J. F. MINER:

My Dear Sir:—I sent to your Journal a day or two since, a copy of the Transactions of the State Medical Society for 1864. I hope you will notice the same in the next number of the Journal. I think it is an excellent volume and worthy of the Society from which it emanates.

Please notice on page 450 that Erie County Medical Society was not represented, nor the University of Buffalo, at the last meeting. Can you not do something, say something, that will inspire both institutions to a representation next year?

In haste, very truly yours,

S. D, WILLARD."

In reviewing the book, or rather in giving a brief account of its contents we say, "Erie County Medical Society was not represented at the last meeting of the State Society, or the University of Buffalo." The following polite note from Dr. Lee explains itself, and we are most happy to make the correction:

"BUFFALO, December 10, 1864.

J. F. MINER, M. D., Editor Buffalo Medical and Surgical Journal :

Dear Sir:—My attention has been called to an editorial article in the November number of your excellent Journal, in which it is stated that the "University of Buffalo was not represented at the last meeting of the New York State Medical Society." It is further stated that "the delegate chosen to represent it was probably detained by 'severe sickness,' for there can be no doubt that nothing but severe *indisposition* could have prevented the duly appointed delegate from discharging his duties," etc.

Now I am happy to inform you that I was the regularly appointed delegate on the part of the Faculty of the University, that I was present at the meeting of the State Society at its last meeting, and offered on behalf of the Faculty a resolution in favor of having a State Board of Examiners appointed to examine all candidates for the degree of Doctor in Medicine; which resolution was also published in your Journal for March last, as having been offered by myself. You may also recollect my reporting the result at the dinner of the Faculty at the last Annual Commencement, at the American Hotel, at which you were present.

As the Faculty of the University has been the first medical incorporate body in this country, to rise above selfish, pecuniary considerations, and take the initiative in an attempt to separate teaching and licensing, or the conferring of medical honors, it hardly seems to me just to ignore even a representation at the meeting where this important action was taken.

Very Respectfully,

CHARLES A. LEE, M. D.,
Late delegate to State Med. Soc. from Buffalo University."

"MEDICAL SOCIETY OF THE STATE OF NEW YORK,
Albany, N. Y., December 13, 1864.

DR. J. F. MINER:

My Dear Sir:—Your favor of the 12th is just received, wherein I am called on to show why the University of Buffalo was not represented by a delegate at the last meeting of this Society. This Society has a register, and has had for many years, wherein each permanent member or delegate records his name. The headings are as follows:

| Name. | Permanent Member, Delegate or Invited. | Represents Medical Society of or College. | Post Office. |
|---|---|---|---|
| Charles A. Lee, | Per. Member, | | Peekskill, N. Y. |

At the meeting of 1864, the one hundred and twenty-fifth name recorded is that of Charles A. Lee, and I have copied precisely as it is in the register. It is from this register that I compile the representation of the Society for the minutes as found on pages 449, 450, 451. Dr. Lee is recorded a Permanent Member, just as he is in 1861 and 1862, where he is registered precisely as he was in 1864. The error lies just here: Dr. Lee omitted to fill the third space, "University of Buffalo," by which the fact would have appeared that he was a delegate from that institution.

The fact that he failed to do this leaves the record just as he made it, that he came as a Permanent Member, his residence being from Peekskill. It does not show that he had any relation to the University or any other institution. If gentlemen fail to mention that they represent a Society, the records will not show that the institution was represented, as it is not. The omission was on the part of Dr. Lee, and except for his information in your letter the fact of his being a delegate would never have come to light.

The case being now understood, the effectual remedy will be in seeing that the University is recorded as represented at the next meeting, and right under it a full record from Erie County Medical Society also.

The fact that Dr. Lee presented measures on behalf of the Faculty of the University, (most commendable measures,) does not even imply that he was a delegate from the University, since in his capacity as permanent member he could advocate that measure, as one of the Faculty of the College, in which position I supposed he acted.

I take pleasure in making this explanation. I should be sorry to do injustice towards the University, or towards the members from Erie, and to do them injustice would be not only far from my heart, but the last way to win their full attendance at our meetings.

Very sincerely yours, S. D. WILLARD.

ALBANY, December 15, 1864.

DR. J. F. MINER:

Dear Sir:—Since my letter of the 13th I have looked over and examined the credentials of delegates from the various medical institutions of the State, to the State Medical Society, as presented at its last meeting and duly filed by the "committee on credentials," at the meeting. There is no credential from the University of Buffalo on file. I am obliged to conclude therefore that my excellent friend Dr. Lee, did not present any credential as delegate from the University, even if he had it in his pocket.

The omission of this formality, as also to register, as delegate, left the University unrepresented, excepting at head and heart, where I know Dr. Lee has the interests of the whole profession and medical science generally. Your position in the Journal was right, and your authority correct.

Truly yours, S. D. WILLARD.

We are not only very glad to have Prof. Lee and Dr. Willard explain the matter of representation, but also very glad to have these resolutions mentioned—most happy to hear from them. We have long desired to know what had become of resolutions which looked so disinterested and self-sacrificing. The last heard was at the Faculty dinner when they were complimented, toasted and praised, we hoped, not spoiled. These resolutions, if adopted and acted upon, would have been worth something to the profession—would have constituted a step of real progress in the standard of professional qualification. We predicted at the time that they would prove idle words, and we now expect that future generations will repeat them as original. This does not in the least detract from their merit, or from the just credit of proposing and introducing them; it only shows that the present age is not ripe for them, that they are in spirit a generation or two in advance of the times in which we live. Dr. Lee in his ripe experience—in his extensive observation, both at home and in other countries, had conceived what would be a great protection to our profession, would elevate its character, increase its real worth, and insure the respect and confidence which it should receive; but his efforts for good will be unavailing while the present active competition exists in the business of manufacturing Doctors; indeed they are made, not so much for what they are worth when graduated, as for the reflex influence upon those who educate and graduate them.

Our zeal for the said resolutions has led us quite off the subject of representation of the County Society, and University, in the State Medical Society, and we return only to say, that if the County Society is of any positive advantage in any respect, it must be in forming connection with the State Society, which is an active and valuable medical organization. In choosing delegates it should select those who will act, or if from any circumstances they cannot, who will resign, that others may be elected, who can attend.

